# Involvement of *Fusobacterium nucleatum* in malignancies except for colorectal cancer: A literature review

**DOI:** 10.3389/fimmu.2022.968649

**Published:** 2022-08-17

**Authors:** Zhixing He, Wei Tian, Qichun Wei, Jing Xu

**Affiliations:** ^1^ Institute of Basic Research in Clinical Medicine, College of Basic Medical Science, Zhejiang Chinese Medical University, Hangzhou, China; ^2^ Department of Breast Surgery, The Second Affiliated Hospital, Zhejiang University, College of Medicine, Hangzhou, China; ^3^ Department of Radiation Oncology, The Second Affiliated Hospital and Cancer Institute (National Ministry of Education Key Laboratory of Cancer Prevention and Intervention), Zhejiang University School of Medicine, Hangzhou, China

**Keywords:** *Fusobacterium nucleatum*, cancer, Gal-GalNAc, prognosis, oral and head and neck cancer

## Abstract

*Fusobacterium nucleatum* (*F. nucleatum*) is originally an oral opportunistic pathogen and accumulating evidence links the presence of *F. nucleatum* with the pathogenicity, development, and prognosis of colorectal cancer (CRC). However, only limited preliminary data is available dealing with the role of *F. nucleatum* in other malignancies except for CRC. The present review aims to update and systematize the latest information about the mechanisms of *F. nucleatum*-mediating carcinogenesis, together with the detection rates, clinicopathological, and molecular features in *F. nucleatum*-associated malignancies. Comparing with adjacent non-tumorous tissue, previous studies have shown an overabundance of intratumoural *F. nucleatum*. Although the prognostic role of *F. nucleatum* is still controversial, a higher prevalence of *F. nucleatum* was usually associated with a more advanced tumor stage and a worse overall survival. Preliminary evidence have shown that epithelial-to-mesenchymal transition (EMT) and relevant inflammation and immune response aroused by *F. nucleatum* may be the probable link between *F. nucleatum* infection and the initiation of oral/head and neck cancer. Further studies are needed to elucidate the etiologic role of the specific microbiota and the connection between the extent of periodontitis and carcinogenesis in different tumor types. The mechanisms of how the antibiotics exerts the critical role in the carcinogenesis and antitumor effects in malignancies other than CRC need to be further explored.

## Introduction

Over 100 trillion bacteria inhabit the human body (1) and the composition of human microbiome is closely associated with the risk of cancer (2). Approximately 20% of total cancer incidences are attributable to bacterial and viral infections, with the primary focus being on the carcinogenic role of *Helicobacter pylori* (*Hp*) in gastric cancer and the human papilloma virus (HPV) in cervical cancer (3). A better understanding of the mechanisms and contribution of the pathogenic microbiota to chronic inflammation and initiation of malignancy may aid the development of novel approaches to the prevention and treatment of cancer.

*Fusobacterium nucleatum* (*F. nucleatum*) is a non-spore-forming, anaerobic, gram-negative, pro-inflammatory bacterium, and primarily inhabits the human oral cavity (4). It has been recognized that *F. nucleatum* acts as an opportunistic pathogen in multiple inflammatory diseases, including periodontitis (5), inflammatory bowel disease (6), liver abscesses (7), rheumatoid arthritis (8), and chorioamnionitis (9). Its involvement in multiple systemic conditions has also been found, such as Alzheimer’s disease (10), ruptured cerebral aneurysm (11), atherosclerosis (12), cardiovascular diseases, adverse pregnancy outcomes, gastro-intestinal disorders, and diabetes (13). Meanwhile, *F. nucleatum* has been initially considered as a bridging organism in the assembly and architecture of polymicrobial biofilms, which has prompted the hypothese that *F. nucleatum* contributes to cancer development (14).

Landmark publication from two independent groups reported an overabundance of intratumoural *F. nucleatum* in colorectal cancer (CRC) tissues, comparing with adjacent non-tumorous mucosa by metagenomic analysis (15, 16). A meta-analysis indicated that the DNA of *F. nucleatum* was higher in CRC tissue, as well as in colorectal polyp tissue compared with adjacent healthy tissue from controls (17). *F. nucleatum* was also more abundant in fecal samples from CRC patients in comparison with healthy controls or individuals with premalignant lesions of the colorectum (17).

Interestingly, Komiya et al. identified the identical strains of *F. nucleatum* in the oral cavity and the CRC tissues from the patients, thus suggesting the oral dissemination of the *F. nucleatum* and its potential role in CRC carcinogenesis (18). Furthermore, *F. nucleatum* could be detected from stages 0 to IV, and no significant differences in the detection rate of *F. nucleatum* could be found among each CRC lesion site from 8 patients, indicating that *F. nucleatum* might adhere to CRC tissue from an early stage of tumorigenesis (19). The elevated level of *F. nucleatum* DNA in CRC tissues has been linked to certain molecules and cell functions, including the microsatellite instability, CpG island methylator phenotype, hMLH1 methylation, and genetic mutations in BRAF and TP53 (20, 21).

Several studies have explored the mechanisms by which *F. nucleatum* exerted its pathogenic roles in CRC. *F. nucleatum* can express a novel bacterial cell surface adhesin protein (FadA), which binds to E-cadherin, triggers β-catenin signaling pathway, consequently stimulates the production of inflammatory cytokines and chemokines and finally drives CRC cell proliferation in *in vitro* and *in vivo* models (16, 22, 23). Another possible etiological factor is that *F. nucleatum* can modulate the tumor-immune microenvironment and exert immunosuppressive activity by impairing natural killer cell and T cell functions (16, 24). *F. nucleatum* is also capable of inducing apoptosis cell death in the peripheral mononuclear blood cells and the polymorphonuclear neutrophils (25). *F. nucleatum* has been associated with a lower density of CD3^+^ T cell, and the secretion of immune cytokines in CRC (16, 26, 27). Furthermore, hydrogen sulfide, a metabolite of *F. nucleatum*, can generate reactive oxygen species, induce DNA damage, and cause single-nucleotide mutations. Hence, *F. nucleatum* may promote oncogenesis by acting as a DNA-damaging agent (28). *F. nucleatum* can also trigger the production of matrix metalloproteinase-9 (MMP-9) and MMP-12, which are important factors for the tumor proliferation, invasion and metastasis in epithelial cells (29). Collectively, the potential of *F. nucleatum* to act as a carcinogen is credible, as it has been shown to activate cell proliferation, induce chronic inflammation and suppress local immune responses.

Strikingly, a high prevalence of *F. nucleatum* in CRC was associated with the poor overall survival (30). Meanwhile, intratumoral *F. nucleatum* was more abundant in CRC tissues of patients who experienced recurrence post chemotherapy compared with those who did not exhibit tumor recurrence (31). However, the relationship between *Fusobacterium*’s abundance and the clinical outcome of CRC has been inconclusive. Two other studies found no relevance between *Fusobacterium* and the prognosis of CRC patients (15, 32). This discrepancy may attribute to the differences in patient cohorts, the methods used for assessing *F. nucleatum*, and chance variations between independent researches.

Overall, there has been extensive research demonstrated that the presence of *F. nucleatum* is associated with the pathogenicity, development, and prognosis of CRC. Epidemiological evidence and molecular mechanism studies have proved a positive association between preexisting inflammatory lesions, such as periodontitis and cancer risk (33). Given that *F. nucleatum* is originally an oral opportunistic pathogen, however, only limited preliminary data is available dealing with the role of *F. nucleatum* in other types of cancer, including esophageal, gastric, or pancreatic cancer, etc. It remains unclear whether *F. nucleatum* exerts a similar oncogenic effect on malignant tumor types except for CRC. The present review aims to update and systematize the latest information about the potential involvement of *F. nucleatum* in carcinogenesis and the significance of *F. nucleatum* as a prognostic and predictive biomarker to anti-tumor therapy in other types of malignancies.

## *F. nucleatum* in oral and head and neck cancer

Human Oral Microbiome Database (www.homd.org) reported the existence of over 700 bacterial strains in human oral cavity (34). The oral bacterial plays an essential role in maintaining the healthy physiological environment in oral cavity and the periodontitis has been identified as an independent risk factor of oral cancer development (35). It has been concerning whether the infection of *F. nucleatum*, a well-known oral bacterium involved in the formation of typical dental plaque on human teeth, may cause oral cancer.

### *F. nucleatum* is overabundant in oral and head and neck cancer

In 1998, the first association study by Nagy et al. found that the level of *Fusobacterium* were significantly higher in oral squamous cell carcinoma (OSCC) than in healthy mucosa (36). The DNA of saliva microbiome isolated from oropharyngeal squamous cell carcinoma (OPSCC), OSCC patients and normal epithelium controls were compared using 16S rRNA amplicon sequencing, to characterize the compositions of saliva microbiota and examine their abundance before and after surgical treatment. The analyses identified a total of 13 assigned phyla present, with 5 of these dominating across all of the samples: *Fusobacteria*, *Proteobacteria*, *Bacteroidetes*, *Actinobacteria*, and *Firmicutes* (37). In another study, cancer lesion samples and anatomically matched normal samples were obtained from the same patients to unravel the connections underlying oral bacterial dysbiosis and OSCC. Bacterial dysbiosis was observed within OSCC surface lesion samples, and drastic changes in surface bacterial communities of OSCC was observed. In particular, *Fusobacterium*, which belongs to periodontitis-correlated taxa, was found to be significantly enriched in OSCC samples. Additionally, several operational taxonomic units belonging to *Fusobacterium* were inferred to be highly involved in OSCC and demonstrated good diagnostic power (38). Consistently, two other studies (39, 40) also confirmed the significantly higher abundance of *F. nucleatum* in swabs of OSCC lesion surface compared to those of normal mucosa from the same patients (**Table 1**).

**Table 1:** Detection rates of *Fusobacterium* spp. in malignancies except for colorectal cancer from previous studies.

Type of cancer	Name of bacteria	Type of Sample	Detection method	Detection rate, %(no. of cases)		Refs.
				Tumor tissue	Normal tissue
OSCC	*Fusobacterium*	Frozen tissue	16S rRNA	10.98 (50)	3.27 (50)	Zhang et al. (40)
OSCC	*F. nucleatum*	Saliva	16S rRNA	0.84 (138)	0.43 (151)	Hsiao et al. (41)
Oral/head and neck cancer	*Fusobacterium*	A meta-analysis of 17 publications		16	10	Bronzato et al. (42)
HNSCC	*Fusobacterium*	Frozen tissue	qPCR	75.9 (74)	–	Desai et al. (43)
OSCC	*F. nucleatum*	Frozen tissue	qPCR	82.1 (151)	–	Neuzillet et al. (44)
HNSCC	*F. nucleatum*	Frozen tissue	qPCR	55.8 (68)	–	Chen et al. (45)
OSCC	*F. nucleatum*	Frozen tissue	PCR	74.0 (50)	26.0 (30)	Mandal et al. (46)
Esophageal cancer	*F. nucleatum*	FFPE	qPCR	23.0 (325)	–	Yamamura et al. (47)
ESCC, GCA	*Fusbacteria*	Frozen tissue	16S rRNA	ESCC, 3.9 (67); GCA, 1.9 (36)	ESCC, 1.7 (67); GCA, 0.3 (36)	Shao et al. (48)
ESCC	*F. nucleatum*	FFPE	qPCR	69.4 (98)	–	Li et al. (49)
ESCC	*F. nucleatum*	FFPE	RNAscope	34.6 (246)	3.3 (246)	Zhang et al. (50)
ESCC	*F. nucleatum*	FFPE	qPCR	29.0 (120)	–	Liu et al. (51)
Esophageal cancer; gastric cancer	*F. nucleatum*	FFPE	qPCR	Esophageal cancer 20 (20); gastric cancer 10 (20)	Esophageal cancer 5 (20); gastric cancer 0 (20)	Yamamura et al. (52)
Gastric cancer	*F. nucleatum*	FFPE	qPCR	26 (80)	2.5 (40)	Araujo et al. (53)
*Hp*-positive gastric cancer	*F. nucleatum*	Frozen tissue	Nested PCR	31.7 (60)	–	Hsieh et al. (54)
Gastric cancer	*F. nucleatum*	Frozen tissue	qPCR	28.75 (80)	23.08 (78)	Boehm et al. (55)
Pancreatic cancer	*Fusobacterium* species	FFPE	qPCR	8.8 (283)	28.0 (25)	Mitsuhashi et al. (56)
Breast cancer	*F. nucleatum*	FFPE	16S rRNA	30.0 (50)	–	Parhi et al. (57)
Bladder cancer	*F. nucleatum*	FFPE	16S rRNA	26.2 (42)	–	Popović et al. (58)

OSCC, oral squamous cell carcinoma; GCA, gastric cardia adenocarcinoma; PCR, polymerase chain reaction; qPCR, quantitative polymerase chain reaction; *Hp, Helicobacter pylori*; *F. nucleatum, Fusobacterium nucleatum*; FFPE, formalin-fixed paraffin-embedded; HNSCC, head and neck squamous cell carcinoma; ESCC, esophageal squamous cell carcinoma.

Since *F. nucleatum* is a bridge organism in the dental plaque and can influence other microorganisms in the oral cancer environment, some studies tried to investigate the correlation between *F. nucleatum* and other microorganisms in oral and head and neck cancer (38, 59–61). The oral bacterial DNA obtained from 20 fresh OSCC biopsies (cases) and 20 deep-epithelium swabs (matched control subjects) was sequenced to characterize the composition of bacterial species as well as the potential function of the bacteriome associated with OSCC. At the species level, this study provided the first epidemiological evidence ever for the association of *F. nucleatum* and *Pseudomonas aeruginosa* with OSCC, substantiating existing evidence on its carcinogenicity. At the subspecies level, some variations in the carcinogenicity of *F. nucleatum* were also suggested (60). *F. nucleatum* and *Porphyromonas gingivalis* existed at higher levels in tumor tissues than in normal tissues, while *Streptococcus sanguinis* was more frequent in normal tissues (61). The association between oral bacterial profile and the risk of OSCC was investigated in another case–control study based on 138 OSCC cases and 151 controls. Three species of periodontopathogenic bacteria, *Prevotella tannerae*, *F. nucleatum*, and *Prevotella intermedia*, were positively associated with the risk of OSCC. Every 1% increase in the total percentage of the above three oral periodontopathogenic bacteria was associated with the 28% increase in the risk of OSCC (OR = 1.28, 95% CI: 1.13–1.44) (41). These results were in accordance with a meta-analysis, which showed an increased prevalence of 6% of *Fusobacterium* in tumor lesions than in non-tumor lesions and a 2.93-fold higher chance of *Fusobacterium* being present in tumor lesion (95% CI, 1.47-5.81) (42).

### The clinicopathologic characteristics associate with *F. nucleatum*

In addition, the abundance of *F. nucleatum* significantly varied by the tumor stage and was related with the prognosis of oral/head and neck cancer patients. An increased oral relative abundance of *F. nucleatum* was observed in the stage III disease of HPV+ OPSCC patients (*p* < 0.05) (62). Another pilot study analyzed the relationship between microbial diversity and the different OSCC stages. At the genus level, *Acinetobacter* and *Fusobacterium* were found being predominant in the late stage of OSCC (63).

A recently published study identified a significant prevalence of *Fusobacterium* in head and neck cancer comparable to CRC, from the genomic dataset of Indian origin. It was worth noting that *F. nucleatum* was related with the presence of extracapsular spread, the potential of invasion and metastasis, and poor survival in early-stage HPV-negative tongue cancer. Furthermore, a genomic landscape of pathogens identified the mutual exclusivity between *F. nucleatum* and HPV in head and squamous cell carcinoma (SCC). *Fusobacterium*-high subgroup of head and neck tumors was associated with an inflamed and pro-tumorigenic microenvironment (43). Hsueh et al. also verified that the high abundance of *F. nucleatum* in laryngeal squamous cell cancer (LSCC) was associated with poor prognosis (64). In contrast, Neuzillet et al. (44) and Chen et al. (45) found that the enrichment of *F. nucleatum* in oral/head and neck SCC cohort was significantly associated with a lower tumor stage, improved overall survival, relapse-free survival, and metastasis-free survival. *F. nucleatum*-positivity was also more frequent in older patients lacking the traditional risk factors of alcohol and smoking. Although these findings were unexpected given its association with poor prognosis in other cancer types, particularly in CRC (65), it was further verified that tumors with high *F. nucleatum* loads displayed low RNA levels of OX40 ligand (TNFSF4) and fibroblasts (PDGFRβ) (44), which predicted the favorable prognosis (66).

In conclusion, *F. nucleatum* is more abundant in oral/head and neck cancer samples than non-cancer samples, highlighting the importance of further research on the possible contribution of *F. nucleatum* to the development of oral/head and neck cancer (**Table 2**). The correlation between the abundance of *F. nucleatum* and the clinical stage of oral/head and neck cancer is conflicting. In contrast with the previous studies, *F. nucleatum* was suggested to be associated with “permissive” tumor microenvironment with low Toll-like receptor 4 (TLR4) signaling and M2 macrophage infiltration in OSCC (44). Further prospective explorations of unique microbial signature in oral/head and neck cancer may facilitate the use of oral bacteria as the biomarker for disease prevention, screening and response evaluation.

**Table 2:** Summary of publications showing the associations between clinicopathological features and *F. nucleatum* positivity in malignancies except for colorectal cancer.

Type of cancer	Samples	Associations	Refs.
OSCC	Saliva	More cigarette smoking, no use of dental floss	Hsiao et al. (41)
HPV+ OPSCC	Saliva, swabs and stool	Stage III disease	Oliva et al. (62)
OSCC	Frozen tissue	Later tumor stage	Zhang et al. (63)
HNSCC	Frozen tissue	Poorer survival and higher extracapsular spread among tongue cancer patients	Desai et al. (43)
LSCC	Frozen tissue and FFPE	Poorer survival	Hsueh et al. (64)
OSCC	Frozen tissue	Older age, less alcohol, less frequent lymph node invasion; better OS, RFS and MFS	Neuzillet et al. (44)
HNSCC	Frozen tissue	Non-smokers, lower tumor stage, better CSS and RFS	Chen et al. (45)
Esophageal cancer	FFPE	Later tumor stage and shorter CSS	Yamamura et al. (47)
ESCC	FFPE	Shorter RFS and poorer chemotherapeutic response	Yamamura et al. (67)
ESCC	Frozen tissue	Later tumor stage	Shao et al. (48)
ESCC	FFPE	Later pT stage, tumor stage and a higher risk of metastasis	Li et al. (49)
ESCC	FFPE	Male, smoker, alcohol, poorer differentiation, later tumor stage, and shorter survival	Zhang et al. (50)
ESCC	FFPE	Poorer chemotherapeutic response	Liu et al. (51)
Gastric cancer	FFPE	Older age, tumor size>4 centimeter	Araujo et al. (53)
*Hp*-positive gastric cancer	Frozen biopsies	Female, later tumor stage, and shorter survival	Hsieh et al. (54)
Gastric cancer	Frozen tissue	Older age and shorter OS in patients with Lauren’s diffuse type	Boehm et al. (55)
Pancreatic cancer	FFPE	Tumor Fusobacterium species status is associated with shorter survival	Mitsuhashi et al. (56)
Cervical cancer	FFPE	Poorer differentiation; shorter OS and PFS	Huang et al. (68)
Lung cancer	Bronchoalveolar lavage	A reduction of F. nucleatum in the responders post anti-PD-1 treatment	Chu et al. (69)

OSCC, oral squamous cell carcinoma; OPSCC, oropharyngeal squamous cell carcinoma; HNSCC, head and neck squamous cell carcinoma; LSCC, laryngeal squamous cell cancer; ESCC, esophageal squamous cell carcinoma; RFS, recurrence free survival; MFS, metastasis free survival; OS, overall survival; CSS, cancer-specific survival; FFPE, formalin-fixed paraffin-embedded; PFS, progression-free survival; *Hp, Helicobacter pylori*; HPV, human papillomavirus; F. nucleatum, Fusobacterium nucleatum; *F. nucleatum, Fusobacterium nucleatum*.

### Carcinogenesis mechanisms of *F. nucleatum* in oral and head and neck cancer

Although the detailed carcinogenesis mechanism of *F. nucleatum* is still unclear, *F. nucleatum* have been suggested to be associated with DNA damage, cell adhesion, epithelial-to-mesenchymal transition (EMT), inflammatory host response, and cell cycle in oral cancer (70, 71). *F. nucleatum* could cause cellular DNA damage by increasing upregulation of γH2AX, and promote cell proliferation *via* the Ku70/p53 pathway in oral cancer cells (70). In addition, by using immunohistochemistry, Mandal et al. provided the first study to propose that the carcinogenesis of *F. nucleatum* in OSCC may be CCL20-related, since there was a significant difference in the prevalence of CCL20-positive cell population between *F. nucleatum*-positive and negative OSCC (*P*=0.034) (46). The component of *F. nucleatum* cell wall extract (FnCW), iso-electric focusing (IEF) and beta-defensin inducer peptide (FAD-I), have been shown to stimulate human oral epithelial cells to secrete CCL20 (72). In addition, CCL20 has been shown to contribute to the oral immune response to bacterial infections and might be involved in the initiation and progression of OSCC (73). Meanwhile, Hsiao et al. suggested that periodontopathogenic bacteria might promote the OSCC oncogenesis by inducing inflammation, and the percentage of periodontopathogenic bacteria were positively related with the level of salivary cytokines, interleukin-1β (IL-1β) and IL-2 (41). Infection of gingival epithelial cells with *F. nucleatum* resulted in the translocation of NF-κB into the nucleus and activated NLRP3 inflammasome and caspase-1, following the expression of IL-1β (74). IL-1β has been shown to promote the OSCC carcinogenesis by increasing the proliferation of dysplastic oral cells and stimulating the oncogenic cytokines (75). Meanwhile, bacterial infections could effectively stimulate the dendritic cells to produce IL-2, which would exert its immuno-regulatory functions (76). Functional prediction based on OSCC biopsies and control subjects also showed that the “inflammatory bacteriome” was enriched in OSCC and genes involved in bacterial mobility, flagellar assembly, bacterial chemotaxis and lipopolysaccharides (LPS) synthesis were enriched in the tumors (60).

**Figure 1:**
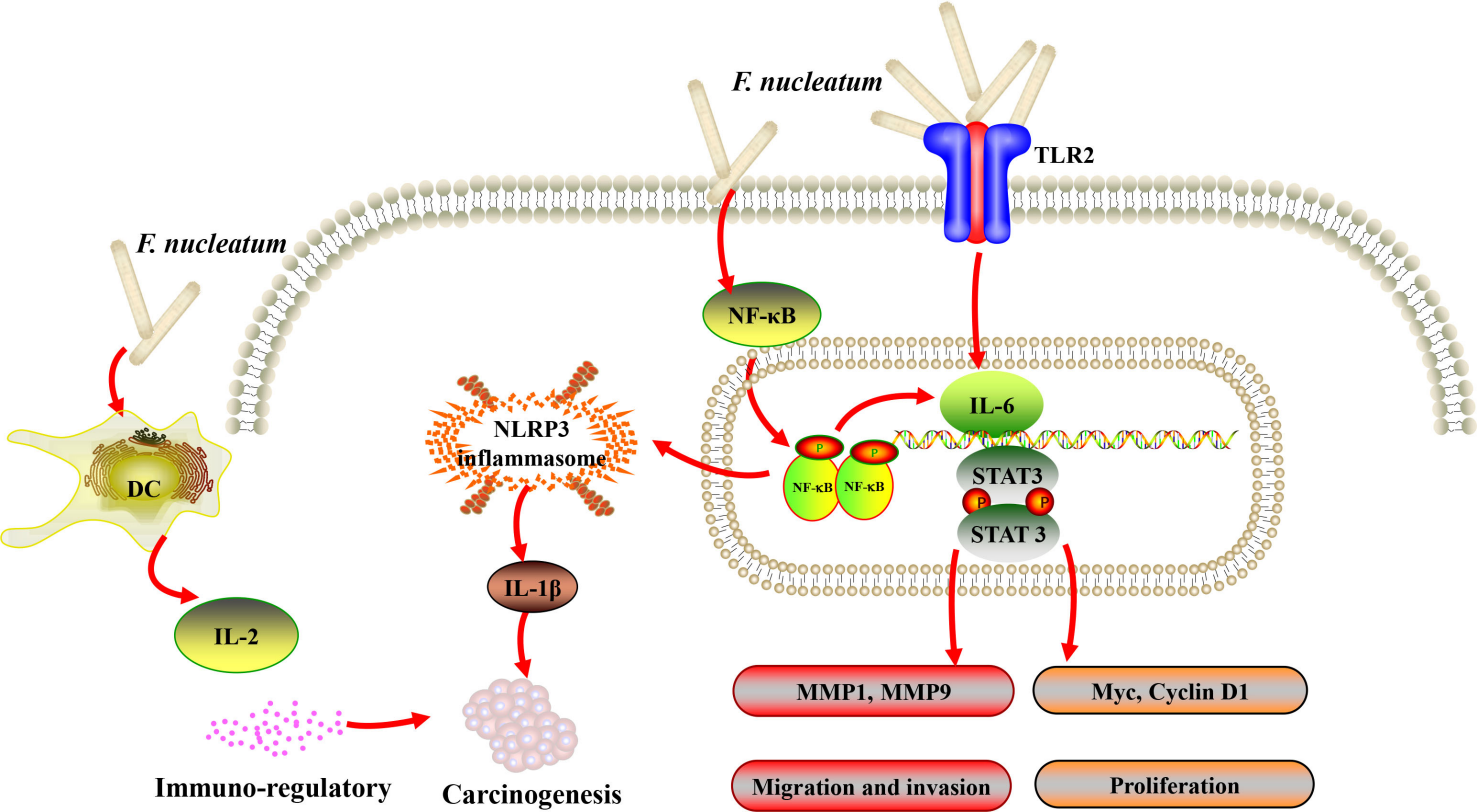
A schematic diagram of the proposed inflammation and immune response aroused by *Fusobacterium nucleatum (F. nucleatum)* in oral and head and neck cancer based on the literature review (41, 74, 76, 77, 80).

Gallimidi et al. provided the first demonstration of a mechanistic role for *F. nucleatum* and *P. gingivalis* in the chemically induced-OSCC tumorigenesis. Co-incubation with *F. nucleatum* and/or *P. gingivalis* profoundly promoted the proliferation of 4-nitroquinoline-1-oxide (4NQO) induced OSCC. Moreover, periodontal pathogens might stimulate tumorigenesis *via* direct interaction with oral epithelial cells, through activating epithelial TLR2 and augmenting signal transducer of IL-6 and activator of transcription-3 (STAT3) axis (77) (**Figure 1**). In the similar 4NQO-induced oral tumor murine model, it was consistent that the mice infected with *F. nucleatum* developed significantly larger and more numerous lesions compared to uninfected controls. Infected oral cancer cells had upregulated expression levels of MMP1, MMP9, and IL-8, the expression of cell survival markers MYC, JAK1, and STAT3, which are implicated in pathways that promote tumorigenesis (80), and EMT markers, ZEB1 and TGF-β were also significantly elevated. Interestingly, Fusobacterial culture supernatant, primarily LPS, was sufficient to induce the expression of IL-8 and MMP, demonstrating that direct contact of the bacteria with cancer cells might not be required to promote carcinogenesis (78).

**Figure 2:**
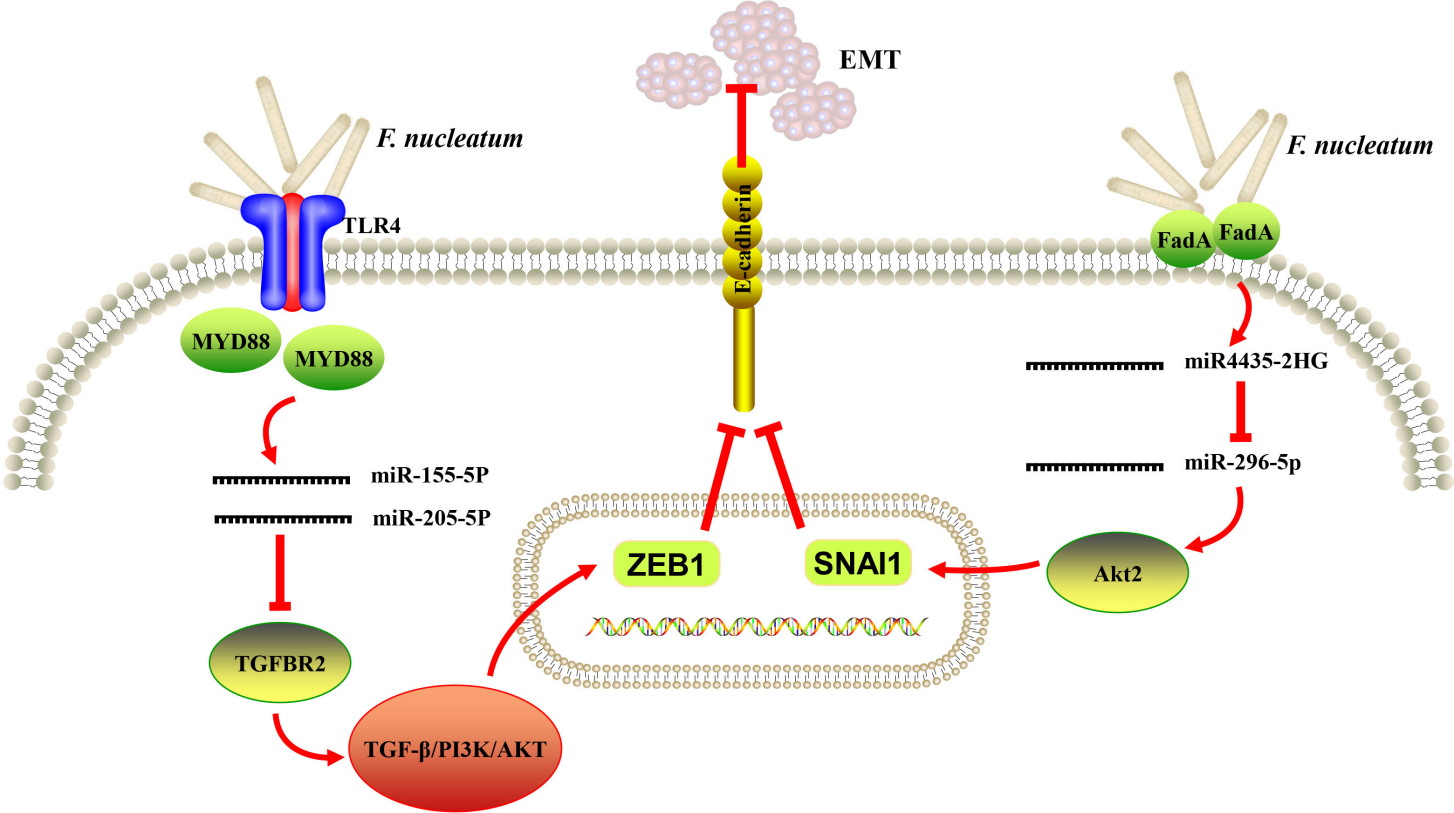
A schematic diagram of the proposed epithelial-to-mesenchymal transition (EMT) mechanism of *Fusobacterium nucleatum (F. nucleatum)* in oral and head and neck cancer based on the literature reviews (64, 78, 79).

It is noteworthy that EMT process has been well described in various carcinomas of epithelial origin and regarded to be associated with initiation and metastasis of tumor (81). EMT was likely to be induced in OSCC cells *in vitro* in response to the stimulation of periodontal pathogens, including *F. nucleatum* and *P. gingivalis* (82). In addition, Zhang et al. analyzed the transcriptome profile of human immortalized oral epithelial cell in response to *F. nucleatum* infection. Tumor-associated genes were integrated, and top 10 potential hub genes (FYN, RAF1, ATM, FOS, CREB, NCOA3, VEGFA, JAK2, CREM and ATF3) were revealed by protein-protein interaction (PPI) network, and LncRNA-hub genes co-expression network comprising 67 dysregulated lncRNAs were generated (83). The same group further reported that *F. nucleatum* infection could eventually trigger EMT in both normal and cancerous oral epithelial cells *via* lncRNA MIR4435-2HG/miR-296-5p/Akt2/SNAI1 signaling pathway (79). A recent study showed that *F. nucleatum* increased the expression of miR-155-5p and miR-205-5p though MYD88-dependent TLR4 signaling, resulting in ethanol metabolism reprogramming *via* suppression of transforming growth factor β receptor 2 (TGFBR2) expression and subsequent suppression of alcohol dehydrogenase 1B (ADH1B) expression and promotion of EMT in LSCC. The positive feed-forward loop between *F. nucleatum* and ethanol metabolism reprogramming finally exacerbated the uncontrolled progression and metastasis of LSCC (64). Taken together, recent data provided preliminary evidence that EMT could be a probable link between *F. nucleatum* infection and the initiation of oral/head and neck epithelial carcinomas (**Figure 2**).

## *F. nucleatum* in esophageal cancer

Several literatures analyzed the relative abundance of *F. nucleatum* in esophageal cancer. *F. nucleatum* DNA was detected in 74 of 325 (23%) resected esophageal cancer specimens, which was significantly more than the matched normal esophageal mucosa. The positive rate of *F. nucleatum* DNA was verified to be significantly associated with tumor stage and shorter cancer-specific survival of esophageal cancer (47). Similarly, high intratumoral *F. nucleatum* burden was also found significantly associated with the poor response to neoadjuvant chemotherapy and had a prognostic significance for predicting poor recurrence-free survival in esophageal squamous cell carcinoma (ESCC) patients (67). Consistent with the previous studies, a positive association between the relative abundance of *Fusobacterium* and the more advanced tumor stage was found in ESCC tissues, by characterizing the microbial communities of paired tumor and normal samples from 67 patients with ESCC in Henan, China (48). In another study from China, the abundance of *Fusobacterium* was increased in tumor tissues and the relative abundance of *F. nucleatum* was closely related to the pT stage and clinical stage of ESCC. The abundance of *F. nucleatum* and tumor mutation burden might be used in combination as a method to predict the potential of metastasis in ESCC, since both a higher mutational burden and *F. nucleatum*-positive was observed in tumors with metastasis than without metastasis (49). However, *F. nucleatum* in the samples of subgingival dental plaque and unstimulated saliva was found not being different between esophageal cancer patients and matched healthy individuals (84). Consequently, the relationship between the abundance of *F. nucleatum* and esophageal cancer is still controversial and the underlying mechanism remains unclear. Clarifying the characteristics of the oral bacteria have potential implications for the early diagnosis in the context of esophageal cancer.

The mechanisms of the involvement of *F. nucleatum* in the esophageal cancer carcinogenesis and chemoresistance were preliminary explored. The top-ranked KEGG pathway in *F. nucleatum*-positive esophageal cancer was the “cytokine–cytokine receptor interaction”, thereby supporting the possible mechanism that *F. nucleatum* might contribute to the aggressive tumor behavior through the activation of chemokines, such as CCL20 (47). The infection and colonization of *F. nucleatum* might also facilitate the immune escape of tumor cells and weaken the antitumor immune response through enriching Treg cells, assisting the long-term self-colonization, and promoting the malignant progression of ESCC (50). In addition, 13 samples of *F. nucleatum*-positive ESCC were analyzed by the whole-exome sequencing and the results showed that the function of the mutant gene was mainly concentrated in the pathways regulating apoptosis and the epidermal growth factor-like protein domain (49). Further analysis revealed that *F. nucleatum* could mediate chemoresistance of ESCC cells by modulating autophagy. In addition, *F. nucleatum* could induce LC3 and ATG7, as well as autophagosome formation to cause chemoresistance against 5-fluorouracil, cisplatin, and Docetaxel. Immunohistochemical studies also confirmed the correlation between *F. nucleatum* infection and ATG7 expression in 284 ESCC specimens (51).

## *F. nucleatum* in gastric cancer

The composition of gastric microbiota is unique and subject to rapid changes caused by the food consumption. The gastric microbiota includes various passenger bacteria undergoing transit from the oral cavity to the lower gut, besides the normal resident bacteria (85). *Hp* was the predominant pathogen in gastric and responsible for the development of gastric cancer (86). According to several preliminary reports, with new sequencing tools, it is increasingly appreciated that not *Hp* alone, but rather the microbiome in whole complexity may trigger a more aggressive oncogenesis (52, 87).

DNA was extracted from 120 gastric fragment samples embedded in paraffin (80 gastric cancer patients; 40 cancer-free patients). *F. nucleatum* was found to be positive in 19 samples (26.0%) of gastric cancer patients, while 1 sample (2.5%) was found to be positive in cancer-free patients. The prevalence of *F. nucleatum* was associated with 11-fold increase in the risk of developing gastric cancer, and was related with advanced age (*P*=0.030) and large tumor size (*P*=0.053) (53). According to the profiles of gastric epithelium-associated microbiota in patients with gastritis, intestinal metaplasia, and gastric cancer, it was indicated that gastric microenvironment was frequently enriched with *Clostridium* and *Fusobacterium* in gastric cancer patients. Furthermore, a receiver operating characteristic curve analysis showed that *Clostridium colicanis*, together with *F. nucleatum*, were potentially considered as the viable diagnostic markers for early diagnosis and positively identified gastric cancer with 100% sensitivity. It also raised the possibility that those bacteria might participate in gastric oncogenesis and the progression of gastric cancer (85). According to another study from southwestern region of Taiwan, *F. nucleatum* was frequently enriched in gastric cancer tissues and positive in 4 samples among the 11 gastric cancer biopsies. The colonization of *F. nucleatum* was able to alter actin filament dynamics to promote the mobility and invasiveness of gastric cancer cells. Additionally, cohort analysis demonstrated that the combined infection of *F. nucleatum* with *Hp* led to a poorer prognosis in gastric cancer patients, indicating the two pathogens acted synergistically to promote the aggressiveness of gastric cancer. Overall, *F. nucleatum* could increase the invasiveness and metastasis of gastric cancer and negatively impact the prognosis of gastric cancer patients (54). *F. nucleatum* was found substantially higher in CRC compared than gastric cancer among a well-characterized cohort of gastric cancer patients. The frequency and bacterial load of *F. nucleatum* were higher in tumorous tissues of CRC and gastric cancer than non-tumorous tissues. Meanwhile, the positivity of *F. nucleatum* was associated with a significantly worse prognosis in Lauren’s diffuse type gastric cancer, but not in the intestinal type gastric cancer patients. As mentioned, *F. nucleatum* may promote carcinogenesis *via* FadA adhesin, which binds to E-cadherin, activates β-catenin signaling and various inflammatory and oncogenic properties of the cells (23). Diffuse type gastric cancer was strongly related with E-cadherin deregulation, which might account for potential molecular mimicry and specific prognostic relevance of *F. nucleatum* to diffuse type of gastric cancer. Further studies are urgently needed to evaluate the possible molecular alterations and therapeutic implications responsible for Lauren’s diffuse type gastric cancer (55).

## *F. nucleatum* in pancreatic cancer

The detection rate of *Fusobacterium* species in 283 patients with pancreatic ductal adenocarcinoma who underwent surgical treatment was 8.8%. Moreover, *Fusobacterium* species status of pancreatic cancer tissue specimens was independently associated with a worse prognosis, suggesting that the presence of *Fusobacterium* species might be related to the malignant potential of pancreatic cancer. *Fusobacterium* species were detected in 28% (7/25) of the paired specimens of normal tissues, using the tumor *Fusobacterium* species-positive cases. Hence, *Fusobacterium* spp. may play a role in the pathogenesis of pancreatic cancer. Further studies are needed to elucidate the roles of these bacteria in the development of pancreatic cancer, which can lead to the development of new diagnostic and therapeutic methods (i.e., eradication) for pancreatic cancer patients (56). Furthermore, circulating plasma and salivary antibodies to *F. nucleatum* were correlated with the severity of intraductal papillary mucinous neoplasms. It was demonstrated that humoral reactivities against *F. nucleatum* were associated with cystic pancreatic neoplasm malignancy (88). Apart from biopsy, a non-invasive method for bacterial analysis may be applied as a diagnostic tool in the future.

## *F. nucleatum* in other non-digestive malignancies

Taken together, except for CRC, *F. nucleatum* has been previously isolated from cancers at other sites along the digestive tract, including the oral, esophagus, stomach, and pancreas. Furthermore, *F. nucleatum* has also recently been implicated in the carcinogenesis and progression of other non-digestive malignancies.

### *F. nucleatum* in breast cancer

Utilizing a permutation test to assess differential taxa of the breast tissue in malignant and benign states demonstrated that the genus *Fusobacterium* was significantly enriched in the breast tissue samples from patients with invasive malignant disease (89). Genomic DNA of *F. nucleatum* was overabundant in human breast cancer. Using two different murine orthotropic models, *F. nucleatum* was found to contribute to breast tumor growth and metastatic progression, most likely through suppressing the accumulation of tumor infiltrating T cells in the tumor microenvironment. *F. nucleatum* could colonize in mammary tumors *via* D-galactose–β(1–3)-N-acetyl-D-galactosamine (Gal-GalNAc), which was overdisplayed on breast cancer cells. Fap2, which was the surface-exposed lectin of *F. nucleatum*, could bind through Gal-GalNAc and mediate breast cancer colonization. Furthermore, antibiotic treatment with metronidazole could counteract *F. nucleatum*-induced breast tumor exacerbation, suggesting that targeting *F. nucleatum* might benefit the treatment of breast cancer (57). *F. nucleatum* promotes CRC progression by activating the TLR4/MyD88 pathway and exhibiting immunomodulatory effects. Whether *F. nucleatum* promote breast cancer progression in a TLR4 dependent manner and through immunomodulation need further exploration (90).

### *F. nucleatum* in cervical cancer

Phylum *Fusobacteria* was predominant in the vaginal microbiota and associated with a high risk of cervical intraepithelial neoplasia in Korea (91). The association between cervical microbiota diversity and the histopathological diagnosis of each stage of cervical cancer was also assessed. Remarkably, *Fusobacterium* spp. was significantly more abundant in the late stages of cervical cancer than in the early stages (HPV-negative or HPV-positive non-cervical lesions) (92). Similarly, there was a distinct high levels of *F. nucleatum* in cervical cancer, especially in relapsed disease. The increased burden of intratumoral *F. nucleatum* predicted correspondingly poorer prognosis in locally advanced stage cervical cancer. Notably, the level of *F. nucleatum* was positively correlated with tumor differentiation, and high burden of intratumoral *F. nucleatum* possessed the characteristic of cancer stem cells. It was proposed that *F. nucleatum* might be one potential cervical cancer diagnostic and prognostic biomarker, and these findings would help to provide a sound rationale and merit for further study of this bacterium (68).

### *F. nucleatum* in bladder cancer and lung cancer

Bacterial communities present in urine samples collected from 12 male patients diagnosed with bladder cancer, and from 11 healthy, age-matched individuals were analyzed using 16S sequencing. Genus *Fusobacterium* was significantly enriched in the bladder cancer group. In an independent sample of 42 bladder cancer tissues, 11 (26%) cases were positive for *F. nucleatum*, detecting by PCR (58).

A metagenomic sequencing analysis on microbial compositions was performed from bronchoalveolar lavage of lung cancer patients who were treated with anti-PD-1 immunotherapy, including 21 non-responders and 19 responders. The relationship between bacterial load and diversity with the clinical response to anti-PD-1 therapy was further analyzed. Airway enriched *Fusobacterium* prior to anti-PD-1 monotherapy was associated with resistance to anti-PD-1 response, providing potential implication in treatment resistance in the immunotherapy of lung cancer (69).

## Discussion

Periodontitis plays an active role in the pathogenesis of human CRC and the rate of new diagnosis of CRC in persons with a positive history of periodontal disease was 1.45 times higher than in those with the negative history after adjusting for a number of potential confounders (93, 94). Upon growing evidences, *F. nucleatum* was shown to be highly abundant in CRC and could be a causative agent of CRC. However, its effects on the development of cancer in other parts of the body have been little studied. We tried to review and enrich almost all the known mechanisms in *F. nucleatum*-mediated carcinogenesis in malignancies except for CRC (**Figures 1**, **2**). The relationships between *F. nucleatum* status and clinicopathological and molecular features in tumor types except for CRC were also reviewed and summarized in **Tables 2**, **3**. Further studies are warranted to fully unravel the intricate regulatory networks of molecular and cellular events underlying the action of *F. nucleatum* in tumorigenesis.

**Table 3:** Reported genes involved in *F. nucleatum*-associated malignancies except for colorectal cancer.

Type of cancer	Associations	Refs.
HNSCC	*Fusobacterium* affected the inflammatory markers IL1B, IL6 and IL8, miRNAs hsa-mir-451a, hsa-mir-675 and has-mir-486-1, and MMP10 in the tongue tumor samples	Desai et al. (43)
LSCC	*F. nucleatum* increased miR-155-5p and miR-205-5p expression to suppress ADH1B and TGFBR2 expression by activating innate immune signaling, resulting in ethanol metabolism reprogramming to allow *F. nucleatum* accumulation and PI3K/AKT signaling pathway activation to promote EMT	Hsueh et al. (64)
OSCC	Low RNA levels of M2 macrophages (CD163), CD4 lymphocytes, fibroblasts (PDGFRβ), TLR4, OX40 ligand (TNFSF4); High levels of TNFSF9 and IL-1β	Neuzillet et al. (44)
HNSCC	Hypermethylation of tumor suppressor genes LXN and SMARCA2	Chen et al. (45)
OSCC	*F. nucleatum* promoted the proliferation ability of Tca8113 by causing DNA damage *via* the Ku70/p53 pathway	Geng et al. (70)
OSCC	The carcinogenesis in *F. nucleatum*-positive OSCC may be CCL20-related	Mandal et al. (46)
Gingival epithelial cells	*F. nucleatum* infection of gingival epithelial cells led to NLRP3 inflammasome-dependent secretion of IL-1β and the danger signals ASC and HMGB1	Bui et al. (74)
OSCC	*F. nucleatum* stimulated tumorigenesis *via* direct interaction with oral epithelial cells through Toll-like receptors	Gallimidi et al. (77)
OSCC	*F. nucleatum* caused a significant increase in cytokines involved in EMT induction and Snail activation	Abdulkareem et al. (82)
Oral epithelial cells	The alteration of lncRNA and potential hub genes in oral epithelial cells in response to *F. nucleatum* infection were revealed.	Zhang et al. (83)
OSCC	*F. nucleatum* promoted EMT through regulation of the lncRNA MIR4435-2HG/miR-296-5p/Akt2/SNAI1 signaling pathway	Zhang et al. (79)
Esophageal cancer	A significant relationship between *F. nucleatum* and the chemokine CCL20 was validated by immunohistochemistry	Yamamura et al. (47)
ESCC	*F. nucleatum* modulates the endogenous LC3 and ATG7 expression, as well as autophagosome formation to induce chemoresistance	Liu et al. (51)
Breast cancer	Occurrence of *F. nucleatum* gDNA in breast cancer samples correlated with high Gal-GalNAc levels and *F. nucleatum* suppressed accumulation of tumor-infiltrating T cells and promoted tumor growth and metastatic progression	Parhi et al. (57)

OSCC, oral squamous cell carcinoma; HNSCC, head and neck squamous cell carcinoma; LSCC, laryngeal squamous cell cancer; ESCC, esophageal squamous cell carcinoma; ADH1B, alcohol dehydrogenase 1B; TGFBR2, transforming growth factor β receptor 2; EMT, epithelial-mesenchymal transition; Gal-GalNAc, D-galactose–β(1–3)-N-acetyl-D-galactosamine; *F. nucleatum, Fusobacterium nucleatum*.

*F. nucleatum* has been primarily characterized as an implicated factor in multi-species biofilms of dental plaque and *F. nucleatum* was enriched in biofilms of OSCC patients (95). By forming bridges between the early and late colonizers, *F. nucleatum* tended to co-adhere with other species in the periodontal biofilms (96). Hence, *F. nucleatum* played a critical role in increasing bacterial diversity of OSCC. Oral hygiene management was beneficial for the reduction of the periodontal biofilm and consequently the amount of *F. nucleatum*, which might contribute to the prevention of OSCC. Although emerging studies suggested that gingivitis and periodontitis, usually caused by microorganisms, could be risk factors for oral/head and neck cancer, it was still difficult to discriminate the concomitant effect or association effect among different pathogenic microorganisms (59). Meanwhile, the extent and severity of periodontitis acted as risk indicators for oral/head and neck cancer even after the adjustments for traditional confound factors (97). Further studies are needed to elucidate the etiologic role of the specific microbiota and the connection between the extent of periodontitis and carcinogenesis in different tumor types.

In addition, the development of biofilm-like structure in the tumor spheroid microenvironment by *F. nucleatum* was also observed in the three-dimensional CRC spheroid model (98). The property of *F. nucleatum* as a bridging organism was demonstrated to be dependent on the host microenvironment in response to alkaline pH (99). Thus, the pathogenicity of *F. nucleatum* in cancer might dependent on pH value influenced by the interplay between the unique microenvironment and microbiome. A low abundance of *F. nucleatum* was found in stomach, which might due to the protective properties of acidic milieu preventing the bacteria dissemination. However, *F. nucleatum* was also not enriched in atrophic gastritis/intestinal metaplasia, where higher pH due to mucosa atrophy was expected. Therefore, further studies will be necessary to address the mechanisms and the appropriate microenvironment of the biofilm formation and development. Clinical studies should also be proposed to explore the microbial profiles and the composition of multiple biofilms during the different stages of tumor evolvement.

In addition to the relevance of *F. nucleatum* with chronic inflammation, *F. nucleatum* has also been suggested to be linked to carcinogenesis and antitumor effect of chemoradiotherapy. *F. nucleatum* enhanced CRC chemoresistance through modulating of TLR4 and MYD88 innate immune signaling, along with specific microRNAs that subsequently activated the autophagy pathway and promoted chemoresistance (31). Furthermore, the colonization of *Fusobacterium* has been traced from primary tumors to liver distal metastases in CRC patients. Treatment of mice bearing a colon cancer xenograft with antibiotic therapy of metronidazole led to a significant decrease in *Fusobacterium* load, cancer cell proliferation and overall tumor exacerbation (100). Similarly, metronidazole could counteract *F. nucleatum*-induced accumulation of tumor infiltrating T cells and growth of breast cancer cells (57). *F. nucleatum* was verified to migrate and locate at the CRC site and metronidazole treatment could cause a lower relative abundance of *F. nucleatum* in both the oral cavity and CRC locus. CRC mice treated with radiotherapy combined with metronidazole had a significantly reduced tumor burden and radiation enteritis (101). High intratumoral F. nucleatum burden predicted a poor response to neoadjuvant chemotherapy in ESCC and airway enriched *Fusobacterium* prior to anti-PD-1 monotherapy was associated with resistance to immunotherapy in lung cancer (47, 67, 69). The mechanisms of how the antibiotics exerts the critical role in the carcinogenesis and antitumor effects in malignancies other than CRC need to be further explored.

Furthermore, previous results have been inconsistent regarding the prevalence and prognostic roles of intratumoral *F. nucleatum*, and the biological interplay between this bacterium and the different tumor immune microenvironment. Inconsistent results among different studies might be due to different ethnic groups (102), methodological variations in terms of technology for detecting *F. nucleatum* (culture, real-time PCR, 16S rRNA metagenomics), the types of samples (biopsy, surface swab or saliva), and the selection of controls (healthy controls or non-cancerous tissues as controls) (103). In addition, the relatively small sample size in the retrospective cohort and case-control studies might also interpret the controversial results. Furthermore, the immune-related gene analysis was only based on selected genes, which were not fully specific of each immune cell subtypes. Overall, further exploration on the mechanisms linking the colonization of *F. nucleatum* with immune microenvironment in different tumor types are urgently needed. The above controversial results also motivate the randomized clinical trials from larger prospective cohorts to verify these findings.

The host polysaccharide Gal-GalNAc, which is overexpressed in CRC and recognized by a microbial protein, Fap2, could trigger *F. nucleatum* binding to the tumor tissues (96). Besides CRC, increasing Gal-GalNAc level was also found in additional various adenocarcinomas of the stomach, prostate, pancreas, ovary, uterus, breast, esophagus, and lung (104). It was demonstrated that oral *F. nucleatum* might probably translocate to the colon *via* the hematogenous route during transient bacteremia, which was frequent in periodontal disease, instead of the gastrointestinal route (57). Its selectivity for Gal-GalNAc-displaying tumors, suggested that additional tumors might be colonized by *F. nucleatum* (57, 96, 104). Therefore, a potential fusobacterial elimination-based cancer therapy may be engineered as a platform for treating high Gal-GaNAc displaying tumors individually in the future (104).

## Conclusions

The current studies shed light on the potential application of *F. nucleatum* as a diagnostic and prognostic biomarker in the context of multiple tumor types. The early periodontal screening, detection and prognosis judgement yield valuable insights into clinical management, which lead to a reduced morbidity and mortality rate and improve the oncologic outcome of cancer patients. In addition, the involvement of periodontal disease in the evolution of some types of cancers through the action of *F. nucleatum* has been proven. Hematogenous *F. nucleatum* can bind and/or invade diverse cell types including oral, colonic epithelial cells, T-cells, keratinocytes and macrophages through its lectin Fap2 (105), which also plays a putative role in carcinogenesis (106). In mice, intravascularly inoculated Fap2-expressing *F. nucleatum* ATCC 23726 specifically colonized mammary tumors, whereas Fap2-deficient bacteria were impaired in tumor colonization. Thus, targeting *F. nucleatum* or Fap2 might be efficient during the treatment of breast cancer (57). There have been multiple studies concerning about anti-cancer therapies through targeting the bacteria, such as vaccination, the change of diet, and the use of probiotics (107–109). This would motivate future research on the mechanisms of the F. nucleatum-initiated cancers for the development of novel approaches to prevent or treat *F. nucleatum*-related diseases.

## Author contributions

ZH, WT, and JX researched data for the article, designed the figures, and wrote the manuscript. QW reviewed/edited the manuscript before submission. All authors approved the final version of the manuscript.

## Funding

This work was supported by the National Natural Science Foundation of China (82074217).

## Conflict of interest

The authors declare that the research was conducted in the absence of any commercial or financial relationships that could be construed as a potential conflict of interest.

## Publisher’s note

All claims expressed in this article are solely those of the authors and do not necessarily represent those of their affiliated organizations, or those of the publisher, the editors and the reviewers. Any product that may be evaluated in this article, or claim that may be made by its manufacturer, is not guaranteed or endorsed by the publisher.
